# A Description of the Statistical Methods for the Vaccine Impact on Diarrhea in Africa (VIDA) Study

**DOI:** 10.1093/cid/ciac968

**Published:** 2023-04-19

**Authors:** Helen Powell, Yuanyuan Liang, Kathleen M Neuzil, Leslie P Jamka, Dilruba Nasrin, Samba O Sow, M Jahangir Hossain, Richard Omore, Karen L Kotloff

**Affiliations:** Center for Vaccine Development and Global Health, University of Maryland School of Medicine, Baltimore, Maryland, USA; Department of Pediatrics, University of Maryland School of Medicine, Baltimore, Maryland, USA; Center for Vaccine Development and Global Health, University of Maryland School of Medicine, Baltimore, Maryland, USA; Department of Epidemiology and Public Health, University of Maryland School of Medicine, Baltimore, Maryland, USA; Center for Vaccine Development and Global Health, University of Maryland School of Medicine, Baltimore, Maryland, USA; Department of Medicine, University of Maryland School of Medicine, Baltimore, Maryland, USA; Center for Vaccine Development and Global Health, University of Maryland School of Medicine, Baltimore, Maryland, USA; Department of Medicine, University of Maryland School of Medicine, Baltimore, Maryland, USA; Center for Vaccine Development and Global Health, University of Maryland School of Medicine, Baltimore, Maryland, USA; Department of Medicine, University of Maryland School of Medicine, Baltimore, Maryland, USA; Centre pour le Développement des Vaccins du Mali (CVD-Mali), Bamako, Mali; Medical Research Council Unit, The Gambia at the London School of Hygiene and Tropical Medicine, Banjul, The Gambia; Kenya Medical Research Institute, Center for Global Health Research (KEMRI-CGHR), Kisumu, Kenya; Center for Vaccine Development and Global Health, University of Maryland School of Medicine, Baltimore, Maryland, USA; Department of Pediatrics, University of Maryland School of Medicine, Baltimore, Maryland, USA

**Keywords:** attributable fraction, attributable episode, matched case-control study, conditional logistic regression, generalized linear mixed models

## Abstract

**Background:**

Diarrheal diseases remain a health threat to children in low- and middle-income countries. The Vaccine Impact on Diarrhea in Africa (VIDA) study was a 36-month, prospective, matched case-control study designed to estimate the etiology, incidence, and adverse clinical consequences of moderate-to-severe diarrhea (MSD) in children aged 0–59 months. VIDA was conducted following rotavirus vaccine introduction at 3 censused sites in sub-Saharan Africa that participated in the Global Enteric Multicenter Study (GEMS) ∼10 years earlier. We describe the study design and statistical methods of VIDA and where they differ from GEMS.

**Methods:**

We aimed to enroll 8–9 MSD cases every 2 weeks from sentinel health centers in 3 age strata (0–11, 12–23, 24–59 months) and 1 to 3 controls matched by age, sex, date of case enrollment, and village. Clinical, epidemiological, and anthropometric data were collected at enrollment and ∼60 days later. A stool specimen collected at enrollment was analyzed by both conventional methods and quantitative PCR for enteric pathogens. For the matched case-control study, we estimated the population-based, pathogen-specific attributable fraction (AF) and attributable incidence adjusted for age, site, and other pathogens, and identified episodes attributable to a specific pathogen for additional analyses. A prospective cohort study nested within the original matched case-control study allowed assessment of (1) the association between potential risk factors and outcomes other than MSD status and (2) the impact of MSD on linear growth.

**Conclusions:**

GEMS and VIDA together comprise the largest and most comprehensive assessment of MSD conducted to date in sub-Saharan Africa populations at highest risk for morbidity and mortality from diarrhea. The statistical methods used in VIDA have endeavored to maximize the use of available data to produce more robust estimates of the pathogen-specific disease burden that might be prevented by effective interventions.

## Teaser

We provide a description of the Vaccine Impact on Diarrhea in Africa (VIDA) study design, data collected, and a detailed explanation of the statistical methods, including where they differ from those of the study predecessor, the Global Enteric Multicenter Study.

Diarrheal disease remains a global health threat for young children. Although mortality and morbidity rates have declined over the past 3 decades, diarrheal diseases are attributed to approximately 500 000 deaths annually among children under 5 years of age [1] and rank as the third highest cause of burden, measured by disability-adjusted life-years, in children younger than 10 years [2]. To continue progress in reducing the burden of diarrheal diseases, contemporaneous estimates of the characteristics of these illnesses are needed using robust methodology to ensure that actionable data are available to inform effective interventions. A series of large epidemiologic studies have been conducted during the past 10 years to assess the pathogen-specific burden of diarrheal disease in different settings, including the community [3] and the hospital [4]. Among these was the Global Enteric Multicenter Study (GEMS; 2007–2011), a 36-month prospective, population-based, age-stratified case-control study of the incidence, etiology, and adverse clinical consequences of medically attended moderate-to-severe diarrhea (MSD) among children aged 0–59 months of age living in 7 censused populations in South Asia and sub-Saharan Africa [5–7]. GEMS-1A (2011–2012), a 12-month follow-on study, included an investigation of the cases of medically attended diarrhea that did not meet the criteria for MSD, termed “less severe diarrhea” [8].

The Vaccine Impact on Diarrhea in Africa (VIDA) study uses comparable clinical and epidemiological methodologies to its predecessors, GEMS and GEMS-1A, to examine the etiology, incidence, and adverse clinical consequences of MSD, post–rotavirus vaccine introduction at 3 of the GEMS sites in sub-Saharan Africa (The Gambia, Mali, and Kenya). Pathogens were detected in stool samples during GEMS, GEMS-1A, and VIDA using “conventional” microbiological methods (culture, immunoassay, multiplex polymerase chain reaction [PCR]), VIDA also routinely used a highly sensitive custom TaqMan Array Card (TAC, Thermo Fisher, Carlsblad, CA, USA) that compartmentalized probe-based quantitative PCR (qPCR) assays; qPCR had been used to a limited extent in a re-analysis of the GEMS results and was not used in GEMS-1A [8, 9]. In this article we discuss the modified analytic strategy that we used to assess the etiology and incidence of MSD during VIDA utilizing the qPCR data and where this approach differs from that of the GEMS re-analysis. We also describe the completely new strategy from that of GEMS for estimating the association between MSD and adverse clinical outcomes.

## STUDY DESIGN

### Scientific Oversight

An International Strategic Advisory Committee (ISAC) was assembled comprising the lead investigators from each site and a multinational group of experts in statistics, global disease burden, gastroenterology and nutrition, demographic surveillance, diarrheal disease epidemiology, rotavirus vaccine efficacy, and enteric microbiology. The ISAC met annually to review the progress of the study and provide guidance on the study design and methodology to ensure key ongoing knowledge gaps in the burden of diarrheal disease among children in low- and middle-income countries were being addressed.

### Sampling Frame for Population-Based Data Collection: The Census, Demographic Surveillance, and Serial Healthcare Utilization and Coverage Surveys

VIDA was conducted, during a 36-month period, at 3 sites in sub-Saharan Africa (Bamako, Mali; Basse and Bangsang, The Gambia; and Siaya County, Kenya). All 3 countries had introduced rotavirus vaccine prior to study commencement: RotaTeq in The Gambia (14 August 2013) and Mali (15 January 2014) and Rotarix in Kenya (1 July 2014).

Each site provided a censused population with an ongoing demographic surveillance system (DSS) that had also been used in GEMS. To ensure the ability to meet sample-size requirements, The Gambia expanded its DSS for VIDA to include Bansang, a neighboring area with similar demography and health indicators. At least twice a year, a Health Care Utilization and Coverage Survey (HUCS) was administered to the primary caretaker of children aged 0–59 months in conjunction with the DSS rounds, similar to the Health Care Utilization and Attitudes Survey (HUAS and HUAS-lite) conducted during GEMS [7].

In Mali and The Gambia, a randomly selected sample of approximately 450 children per age stratum (0–11 months, 12–23 months, 24–59 months) was selected for each round, while in Kenya, all children aged 0–59 months participated in each round of HUCS. The aim was to determine the proportion of children who had an episode of MSD in the preceding 7 days (using an adaptation of the case eligibility criteria) and did not seek care at a sentinel health center (SHC) from which MSD cases were recruited for the case-control study. These data were used to calculate population-based disease incidence estimates derived from children seeking care at the SHCs and adjusted for children who do not seek care at the SHCs for MSD.

### Enumeration of MSD Cases and Enrollment of Matched Cases and Controls to Determine MSD Etiology

The epidemiologic and clinical methods used in GEMS and GEMS-1A have been detailed elsewhere [5, 7, 8]. These publications describe the formative work to select censused populations and the ongoing DSS; MSD case definition; training and oversight; case ascertainment; case and control inclusion criteria; collection of demographic, clinical, and epidemiologic data; clinical examination and anthropometry; stool sampling and processing; use of a memory aid to determine duration of diarrhea; detection of deaths and performance of verbal autopsy; ethical considerations; and analytic methods.

These same epidemiologic and clinical methods were subsequently used in VIDA and are briefly described here. VIDA staff were situated at the intake area of each SHC to detect and enumerate all children aged 0–59 months belonging to the DSS who presented with 3 or more abnormally loose stools in the previous 24 hours. These children underwent eligibility screening and all those who met criteria for MSD were considered eligible. The first 8–9 eligible MSD cases every 2 weeks in each of 3 age strata (0–11 months, 12–23 months, and 24–59 months) who provided informed consent were enrolled, totaling approximately 220 MSD cases per stratum per year or 1980 cases over the 36-month enrollment period. This strategy respected site-capacity limits while allowing even enrollment throughout the year. For each enrolled case, 1–3 children were randomly selected as controls from the DSS database using a computerized algorithm to match by age (plus or minus 2 months for 0–11 months of age and plus or minus 4 months for 12–59 months of age), sex, date of case enrollment (within 14 days), and village. The required number of controls was determined at each site by tracking enrollment in every 2 weeks, using 1:1 case:control matching if 7–9 cases were enrolled, 1:2 matching if 4–6 cases were enrolled, and 1:3 matching if 3 or fewer cases were enrolled. All DSS residents have a unique identification number that was recorded and used to ensure that a case could not simultaneously be enrolled as a control, or vice versa, and to track multiple enrollments for analysis purposes.

### Data Collection at Enrollment

The primary caretaker of each case and control underwent a standardized interview to collect demographic, epidemiologic, and clinical characteristics of the child. Data collected matched GEMS, with minor exceptions mainly related to water and sanitation and breastfeeding. VIDA staff examined the child, measured height (or length for those <24 months of age or those unable to stand), weight, mid-upper arm circumference (MUAC), respiratory rate, capillary refill time, and axillary temperature according to standardized procedures [7]. If a case was hospitalized, the study team documented the child's management and clinical status throughout the stay.

### Memory Aid to Record the Occurrence of Diarrhea for 14 Days After Enrollment

The primary caretaker of cases and controls was provided with a simple pictorial memory aid card at enrollment [7] and underwent training on how to record for a period of up to 14 days the number of days, if any, following enrollment that the child experienced diarrhea (≥3 abnormally loose stools in a 24-hour period). The study team reviewed and collected the memory aid at the follow-up visit 50–90 days post-enrollment.

### Stool Sample Collection and Laboratory Testing

All enrolled cases and controls provided a single, fresh, whole stool specimen within 12 hours of arriving at the SHC to be eligible for inclusion. If a case was to receive antibiotics before passage of a whole stool, a rectal swab was also obtained prior to treatment to enable cultivation of bacterial pathogens. Methods of collection and transport of whole stool and rectal swabs have been described in detail [7].

Conventional methods (bacterial culture, multiplex PCR, reverse transcription [RT]–PCR, and immunoassay) were performed at site laboratories to detect putative bacterial, viral, and protozoan enteropathogens using the uniform methods of GEMS [10]. One difference from GEMS was the use of a modified multiplex PCR to detect diarrheagenic *Escherichia coli* pathotypes, also used in GEMS-1A [8]. Serotyping for *Shigella* was performed at the University of Maryland Center for Vaccine Development and Global Health and phenotypic assays for enterotoxigenic *E. coli* (ETEC) colonization factor antigens were performed at the Universidad de Chile (courtesy of Roberto Vidal).

In addition, during VIDA, each site performed qPCR for all cases and their first matched control, whereas in GEMS, qPCR was performed retrospectively on a random subset of cases and their first matched control [9]. No qPCR was done in GEMS-1A. Also notable is that *Giardia* was omitted during the third year of VIDA due to a supplier error. All remaining cards, which included the *Giardia* probe, were diverted to Kenya where preliminary analysis suggested high rates of *Giardia* positivity. Approximately one-third of participants in Mali and The Gambia had no qPCR data for *Giardia*, although testing by immunoassay continued. A list of all pathogens tested for by qPCR and subsequently included in the analysis is provided in Supplementary Table 1.

Conventional methods of pathogen detection produce a binary result that indicates the presence (or absence) of the pathogen. However, qPCR produces a continuous result in the form of a quantification cycle threshold (Ct), an inverse metric of quantity, where the upper bound of positivity is set at 35 [9]. For the primary analyses, GEMS utilized conventional microbiological methods [5] while VIDA used qPCR.

### Assembling a Nested Prospective Cohort by Performing Single Follow-up Visit to the Household to Assess MSD Outcome

All case and control participants underwent a single follow-up visit at home approximately 2–3 months after enrollment (acceptable range: 50–90 days), which resulted in a new prospective cohort study nested within the original matched case-control study. This cohort was used to examine potential outcomes—namely, vital status and interim growth, and their association with an episode of MSD as compared with controls without diarrhea at enrollment.

### Ethics

This study was approved by the ethical review committees at the University of Maryland, Baltimore (HP-00062472); the Centers for Disease Control and Prevention (CDC; reliance agreement 6729); The Gambia Government/Medical Research Council/Gambia at the London School of Hygiene and Tropical Medicine (1409); the Comité d'Ethique de la Faculté de Médecine, de Pharmacie, et d'Odonto-Stomatologie, Bamako, Mali (no number); and the Kenya Medical Research Institute Scientific and Ethics Review Unit in Siaya County, Kenya (SSE 2996). Informed written consent was obtained from all participants prior to initiation of study procedures.

## STATISTICAL METHODS

### Estimating the Etiology and Incidence of MSD

As in GEMS, the population-based pathogen-specific attributable fraction (AF) and attributable incidence per 100 child-years were estimated. Using a conditional logistic regression (CLR) model, we estimated the association between the quantity of pathogen in a child's stool at enrollment and the child's case or control status: let *y* be an *n* × 1 vector indicating the case or control status of each of the *i* = 1, …, *n* enrolled children, *q* the probability of a child being a case (ie, *q* = Prob [*y* = 1]), and *X* an *n* × *m* matrix of *m* independent explanatory variables, each of which is an enteropathogen. A multiple CLR model can therefore be given by the following equation:


(1) $$\mathrm{logit}(q)=\beta_{0s}+\beta X$$

where β is a vector of *m* regression coefficients associated with each of the *m* enteropathogens and *β*_0*s*_ is the constant term for the *S*^th^ stratum (ie, matching group). The exponential of each β-coefficient is interpreted as the increase (or decrease) in the odds of being an MSD case versus control for every unit increase in pathogen quantity (Ct).

Each of the enteric pathogens assessed by qPCR could potentially be included on the right-hand side of Equation (1). However, pathogens that occur in only a small number of case and control children result in issues of model convergence, particularly when using the bootstrapping method, described below, for estimation of the associated confidence intervals (CIs). We therefore included only pathogens that were identified in at least 2% of all case and control stool samples. Positivity was determined by a Ct below the limit of detection (<35) [9]. We included an interaction term for potential effect modification by study site and separately by age stratum, which was different from that of the GEMS re-analysis, where study site was included as a random rather than a fixed effect [9]. A single CLR model that encompassed all enteric pathogens meeting our criteria and possible interactions could easily become unwieldy and result in overfitting, giving a description of the random error rather than the relationship between the pathogen and case-control status. We therefore created a separate model focusing on the association of a single pathogen with case-control status, termed the pathogen “of interest,” while all other pathogens were included only as potential confounders. Using this approach, the interaction terms for study site and age stratum were only included for the pathogen of interest. We allowed for a more flexible relationship between pathogen quantity and case-control status by including a quadratic term for the pathogen of interest when the associated regression coefficient was statistically significant (*P* < .05).

For each of the *j* = 1, …, *p* pathogens that met our criteria for inclusion. Let *x*_*j*_ be the pathogen of interest and *X*_−*j*_ be a matrix of all the other pathogens. The proposed CLR model was specified as follows:


$$\begin{array}{rl} \mathrm{logit}(q)\;= & \beta_{0s}+\beta_{j}x_{j}+\beta_{\;j2}x_{j}^{2}+\beta_{\;js}x_{j}\;\cdot \;\mathrm{site}+\beta_{\;ja}x_{j}\;\\ & \cdot \;\mathrm{age}\;\mathrm{group}+\beta_{-j}X_{-j} \end{array}$$

where β*_j_* are the coefficients associated with the pathogen of interest *j*. The odds ratio (OR*_j_*) for a 1-unit increase in *x*_*j*_ for pathogen *j* is therefore calculated as follows:


(2) ORj=odds(xj+1)odds(xj)=exp(β0s+βj(xj+1)+βj2(xj+1)2+βjs(xj+1)⋅site+βja(xj+1)⋅agegroup+β−jX−j)exp(β0s+βjxj+βj2xj2+βjsxj⋅site+βjaxj⋅agegroup+β−jX−j)=exp(βj+βj2(2xj+1)+βjs⋅site+βja⋅agegroup)

The case-control study design does not allow for estimation of the probability of MSD among those not exposed to the pathogen of interest. We therefore cannot directly estimate the relative risk (RR) of MSD given exposure. However, if we assume the incidence of MSD in the population is sufficiently small, then OR*_j_* given by Equation (2) can be used as an approximation of the RR*_j_*.

### Population Pathogen-Specific Attributable Fraction of MSD

Methods for estimating an AF from the OR for a binary risk factor are well documented and are easily extended to a categorical exposure [11]. We categorized the continuous exposure by allowing each pathogen Ct value to be a category and the reference value was no pathogen detected (Ct ≥ 35) [9]. The pathogen-specific attributable fraction, AF_*j*_ for pathogen *j*, where pathogen *j* is a categorical exposure, is therefore the sum of the individual AF_*ij*_ for each case child [11]. This is analogous to the more common $AF_{j}=\mathrm{prop}(j|\mathrm{Case})\left(1-\frac{1}{OR_{j}}\right)$, where the AF for the binary risk factor *j* is the proportion of cases for whom the risk factor *j* is present multiplied by 1 minus the reciprocal of the adjusted OR*_j_* for the risk factor *j*. Let AF_*ij*_ denote the AF for case child *i* (*i* = 1, 2, …, *m*) and pathogen *j,* so that the pathogen-specific AF can be expressed as follows, where *m* is the total number of cases:


(3) $$\begin{array}{rl} AF_{j} & =\overset{m}{\underset{i\;=\;1}{\sum }}\;AF_{ij}\\ & =1-\frac{1}{m}\overset{m}{\underset{i\;=\;1}{\sum }}\frac{1}{OR_{ij}} \end{array}$$

The change from a continuous to a categorical exposure means that OR*_ij_* in Equation (3) is now the odds of having MSD for child *i*’s pathogen *j* Ct quantity compared with having none of pathogen *j* detected and is therefore reduced to the following:


$$\begin{array}{rl} OR_{j} & =\frac{\mathrm{odds}(x_{j}=\mathrm{Ct})}{\mathrm{odds}(x_{j}=0)}\\ & =\exp(\beta_{j}+\;\beta_{\;j2}+\beta_{\;js}\cdot \mathrm{site}+\beta_{\;ja}\cdot \;\mathrm{age}\;\mathrm{group}). \end{array}$$

### Pathogen-Specific Attributable Incidence of MSD

To estimate the pathogen-specific AI in the population of MSD per 100 child-years, we use the same approach as in GEMS [6]. For pathogen *j*, the attributable incidence of MSD per 100 child-years is estimated as follows:


(4) $$AI_{j}=\;\frac{AF_{j}M}{3rN}\times 100$$

where AF_*j*_ is estimated by Equation (4), *M* is the total number of children eligible to be enrolled in the VIDA study, *r* is the estimated proportion of children with MSD, derived from the HUCS, seeking care at a SHC [6], *N* is the number of children residing within the DSS, and 3 is the number of study years. Both *N* and *M* are collected as part of the study; *M* is recorded by the study staff stationed at each of the SHCs, and the total number of children residing in the DSS is recorded during each DSS round (*N* is the median over all rounds).

### Estimating Confidence Intervals for the Pathogen-Specific Attributable Fraction and Incidence

There is currently no simple, unified, or generalizable approach to estimating the variance, and thus the standard error, of an AF. We therefore used a bootstrap approach to estimate the 95% CIs for the pathogen-specific AF_*j*_ and AI_*j*_. This approach was also used in the GEMS re-analysis [9]. Bootstrapping requires resampling with replacement from the original data, which allows us to perform computations on the resampled data to create a distribution of estimates without making distributional assumptions about the underlying data and the variance.

We created 5000 bootstrap samples by randomly sampling from the *m* VIDA cases. Each sample will have the same number of cases as the original dataset. For each bootstrap sample we estimated the AF_*j*_ and subsequently the AI_*j*_, using the procedures outlined above, to create a distribution of estimates for each. The 95% CI is taken to be the lower 2.5th and upper 97.5th percentiles of this ordered distribution.

### Assessing the Clinical Consequences of MSD

We examined linear growth faltering using the prospective cohort study nested within the original matched case-control study, expanding upon the methods used in GEMS by performing a longitudinal analysis accounting for potential confounders. The matching, inherited from the original matched case-control study design, was accounted for within our modeling approach. Failure to do so could result in potentially biased results.

We used a linear mixed-effects model, with restricted maximum likelihood, to capture both the correlation in the repeated height-for-age *z* (HAZ) scores measured at enrollment and follow-up and also, separately, the matched case-control sets, treating each set as a cluster of correlated individuals. Let *y* be an *m* × 1 vector of the *y*_*i*,*t*_ HAZ scores, where each child *i* will have 2 measurements *t* = 1, 2, for enrollment and follow-up, and therefore *m* = 2*n* and *n* is the number of enrolled participants. Let *X* be an *m* × *p* matrix of *p* fixed independent explanatory variables and β the *p* × 1 vector of associated coefficients, *Z* an *m* × *q* design matrix for the random effects of the *q* case-control sets, which can vary from 2 to 4 individuals per matched case-control set, and *c* the *q* × 1 vector of associated random effects, and finally, *V* an *m* × *n* design matrix for the random effects of the longitudinal repeated measurements and *l* the *n* × 1 vector of associated random effects:


y=Xβ+Zc+Vl

As the goal is to understand the impact of an episode of MSD on HAZ, we included in the model measures of socioeconomic status, duration of time from enrollment to follow-up, study site, and study age group as potentially confounding variables. We also considered possible interactions between MSD status at enrollment, age group, study site, and duration of time from enrollment to follow-up. Only interactions that were significant at *P* < .1 were retained in the final model. Based on the final linear mixed-effects model, the difference in HAZ between those who had an episode of MSD at enrollment and those who did not at specific times from enrollment to the maximum follow-up time point of 90 days was calculated using Scheffé-adjusted 95% CIs.

### Other Uses of the Prospective Cohort

Depending on the type of the outcome of interest, generalized linear mixed-effects model can be used for any analysis where all enrolled participants would be included but the MSD status of the child at enrollment (case or control status) is not the primary outcome. The inclusion of the second set of random effects would only be necessary if utilizing both the enrollment and follow-up data in the outcome; however, the random effects for the case-control sets should always be considered necessary due to the matched study design and therefore included.

## DISCUSSION

GEMS and VIDA together comprise the largest and most comprehensive assessment of MSD conducted to date in populations at the high risk for morbidity and mortality from diarrhea. A matched case-control design was selected to study MSD, a rare event that does not occur with sufficient frequency to be studied in a longitudinal cohort but represents the episodes of diarrhea that pose the greatest threat to child health.

The approach used in VIDA to estimate the etiology of MSD have evolved from those of GEMS. The methods used to calculate the pathogen-specific AF for a binary risk factor in a case-control setting, as in GEMS, versus a categorical exposure, as in the GEMS re-analysis, have been well established [11]. Treating an initially continuous exposure as categorical is a common approach as it allows a single AF to be estimated, providing a far more easily interpretable result. While we took a very similar approach in VIDA to that of the GEMS re-analysis, we removed the restriction that individual pathogen-specific ORs below 1 would be considered implausible and truncated to null. This seemingly small change can have a substantial impact, particularly when estimating the AF for a pathogen where the association with MSD is not well understood and can often be present in the stool in small quantities. The AF for such a pathogen is pulled towards zero by the ORs below 1 and can even result in a negative overall AF. There is biological plausibility that some putative enteropathogens may lower the risk of acute diarrhea among children in some settings [12], suggesting that it could be reasonable to assume that our understanding of the role of some pathogens within the intestinal microbiome is not well understood and further investigation is warranted.

While the approach of treating a quantitative exposure as categorical is routinely used in analyses and in this scenario has the advantage of producing a single AF estimate that can be readily interpreted, it does have the drawback of requiring the user to select a reference category. It has also been shown that the resulting AF is underestimated. While this is a very real limitation of our analyses, we felt it was necessary to ensure that the results of this study were comparable to those of other studies.

The prospective cohort study nested within the original matched case-control study design provides a unique opportunity to examine the impact of an episode of MSD at enrollment over time, a feature not typically available from a standard case-control study design. The use of the second random-effect component of the analytical method proposed allows for consideration of the inherited clustering of case and control children and limits any bias introduced by the original study design. Despite our approach treating the study as a cohort, it should be noted that children were not being followed to see if they developed the exposure—in this case, MSD. The children enrolled in VIDA were enrolled based on their exposure status at the time of enrollment and therefore may not provide results comparable to those seen with a random sample of all children under the age of 5 years living in sub-Saharan Africa.

In summary, the statistical methods used in the VIDA study have endeavored to maximize the use of the available data to produce more robust estimates of the etiology, incidence, and adverse clinical consequences of MSD in sub-Saharan Africa—the region that continues to report more than half of global deaths among children younger than 5 years [13].

## Supplementary Data

Supplementary materials are available at *Clinical Infectious Diseases* online. Consisting of data provided by the authors to benefit the reader, the posted materials are not copyedited and are the sole responsibility of the authors, so questions or comments should be addressed to the corresponding author.

## Supplementary Material

ciac968_Supplementary_Data

## Notes

***Acknowledgments*.** The authors express their deep gratitude to the families who participated in these studies, the clinical and field staff for their exceptional hard work and dedication, and to the physicians, administration, and health officials at every site who generously provided facilities and support for the conduct of the study. They are grateful to Catherine Johnson, Chris Focht, and Nora Watson at the Emmes Company, LLC, for expert data management and reporting. Special thanks go to Carl Kirkwood, Duncan Steele, and Anita Zaidi at the Bill & Melinda Gates Foundation for helpful oversight, and to the following members of the International Scientific Advisory Committee for providing insightful comments and guidance: Janet Wittes (Chair), George Armah, John Clemens, Christopher Duggan, Stephane Helleringer, Ali Mokdad, James Nataro, and Halvor Sommerfelt. They also thank Jacqueline E. Tate at the Centers for Disease Control and Prevention who assisted in the sample-size calculations used in the study.

***Financial support*.** This work was supported by the Bill & Melinda Gates Foundation grants OPP1111236 and OPP1116751.

***Supplement sponsorship*.** This supplement was sponsored by the Bill & Melinda Gates Foundation. This study is based on research funded by grants from the Bill & Melinda Gates Foundation (OPP1111236/OPP1116751).
