# Supplementary material for: A Description of the Statistical Methods for the Vaccine Impact on Diarrhea in Africa (VIDA) Study
Source: Clin Infect Dis. 2023 Apr 19;76(Suppl 1):S5–S11. doi: 10.1093/cid/ciac968 (PMC10116558; doi:10.1093/cid/ciac968)
Supplement: ciac968_Supplementary_Data [file ciac968_supplementary_data.docx]

**Supplemental: A description of the statistical methods for the Vaccine Impact on Diarrhea in Africa (VIDA) study**

Helen Powell, Yuanyuan Liang, Kathleen M. Neuzil, Leslie P. Jamka, Dilruba Nasrin, Samba O. Sow, M. Jahangir Hossain, Richard Omore, Karen L. Kotloff

1. Supplemental Table S1. Pathogens included on the qPCR TaqMan Array Card (TAC) which were analyzed as part of the VIDA study.

| **Supplemental Table S1. Pathogens included on the qPCR TaqMan Array Card (TAC) which were analyzed as part of the VIDA study.** | |
| --- | --- |
| Adenovirus 40/41 | Norovirus GII |
| *Aeromonas* spp. | *Plesiomonas* spp. |
| Astrovirus | Rotavirus |
| Toxigenic *Bacillus* fragilis | *Salmonella* spp. |
| *Campylobacter* spp. | Sapovirus |
| *Cryptosporidium* spp. | *Shigella* spp./EIEC |
| *Enterocytozoon bieneusi* | Enteroaggregative *E. coli* |
| Giardia | Heat stable- or heat labile- producing enterotoxigenic *E. coli* |
| *Helicobacter pylori* | Enteropathogenic *E. coli* |
| Norovirus GI | Shiga toxin-producing *E. coli* |
